# Overexpression of metastasis-associated *MTA1* mRNA in invasive oesophageal carcinomas

**DOI:** 10.1038/sj.bjc.6690274

**Published:** 1999-04

**Authors:** Y Toh, H Kuwano, M Mori, G L Nicolson, K Sugimachi

**Affiliations:** Division of Pathology, Clinical Research Institute, National Kyushu Cancer Center, Fukuoka, 811-1395, Japan; Department of Surgery II, Faculty of Medicine, Kyushu University, Fukuoka, 812-8582, Japan; Department of Surgery, Medical Institute of Bioregulation, Kyushu University, Beppu, 874-0838, Japan; The Institute for Molecular Medicine, Huntington Beach, CA, 92649, USA

**Keywords:** *MTA1*, oesophageal carcinoma, invasion, metastasis, reverse transcription polymerase chain reaction

## Abstract

The *MTA1* gene is a recently identified novel candidate breast cancer metastasis-associated gene which has been implicated in the signal transduction or regulation of gene expression. We examined the mRNA expression levels of the *MTA1*, the human homologue of the rat *mta1* gene in 47 surgically resected oesophageal squamous cell carcinomas by quantitative reverse transcription polymerase chain reaction. The relative overexpression of *MTA1* mRNA (tumour/normal ratio ≥ 2) was observed in 16 out of 47 (34.0%) oesophageal carcinomas. Oesophageal tumours overexpressing *MTA1* mRNA (T/N ratio ≥ 2) showed significantly higher frequencies of adventitial invasion (*P* < 0.05) and lymph node metastasis (*P* < 0.05), and tended to have a higher rate of lymphatic involvement than the remaining tumours. Thus, the data suggest that the *MTA1* gene might play an important role in invasion and metastasis of oesophageal carcinomas. © 1999 Cancer Research Campaign


*British Journal of Cancer* (1999) **79**(11/12), 1723–1726
© 1999 Cancer Research Campaign
Article no. bjoc.1998.0274

Short communication

### Overexpression of metastasis-associated *MTA1* mRNA in invasive oesophageal carcinomas

**Y Toh[1], H Kuwano[2], M Mori[3], GL Nicolson[4] and K Sugimachi[2]**

[1]Division of Pathology, Clinical Research Institute, National Kyushu Cancer Center, Fukuoka 811-1395, Japan; [2]Department of Surgery II, Faculty of Medicine, Kyushu University, Fukuoka 812-8582, Japan; [3]Department of Surgery, Medical Institute of Bioregulation, Kyushu University, Beppu 874-0838, Japan; [4]The Institute for Molecular Medicine, Huntington Beach, CA 92649, USA

**Summary** The *MTA1* gene is a recently identified novel candidate breast cancer metastasis-associated gene which has been implicated in the signal transduction or regulation of gene expression. We examined the mRNA expression levels of the *MTA1*, the human homologue of the rat *mta1* gene in 47 surgically resected oesophageal squamous cell carcinomas by quantitative reverse transcription polymerase chain reaction. The relative overexpression of *MTA1* mRNA (tumour/normal ratio ≥ 2) was observed in 16 out of 47 (34.0%) oesophageal carcinomas. Oesophageal tumours overexpressing *MTA1* mRNA (T/N ratio ≥ 2) showed significantly higher frequencies of adventitial invasion ($P < 0.05$) and lymph node metastasis ($P < 0.05$), and tended to have a higher rate of lymphatic involvement than the remaining tumours. Thus, the data suggest that the *MTA1* gene might play an important role in invasion and metastasis of oesophageal carcinomas.

**Keywords:** *MTA1*; oesophageal carcinoma; invasion; metastasis; reverse transcription polymerase chain reaction

Of all malignant neoplasms, human oesophageal carcinoma is among the tumours with the poorest prognosis (Sugimachi et al, 1988). Similar to other malignant tumours, this undesirable prognosis in the patients with oesophageal carcinomas has been attributed to invasion and metastasis (Sugimachi et al, 1988). In oesophageal carcinomas, lymph node metastasis and invasion to the neighbouring organs such as the aorta, trachea and bronchus, pericardium and lungs are the most important event in the failure of the treatment of oesophageal carcinomas. Therefore, it is a major goal to identify the molecular mechanisms of invasion and metastasis of the oesophageal carcinomas. Although molecular studies on human oesophageal carcinogenesis have revealed frequent genetic abnormalities including c-*myc* amplification (Lu et al, 1988), epidermal growth factor receptor amplification (Lu et al, 1988), *p53* mutations (Hollstein et al, 1990) and cyclin D1 overexpression (Nakagawa et al, 1995), very little is known about the role of these alterations in the mechanisms of invasion and metastasis of this type of cancer.

Metastasis is a complex series of events that involves several gene products, including those important in the detachment of neoplastic cells from the primary tumour, penetration into the blood and lymphatics, arrest at distant sites by adhesion to endothelial cells and underlying matrix, extravasation, the induction of angiogenesis, evasion of host anti-tumour responses, and the growth at metastatic sites (Nicolson, 1988; Liotta et al, 1991). To identify the genes involved in cancer metastasis, we previously performed differential cDNA library screening using the 13762NF rat mammary adenocarcinoma metastatic system (Pencil et al, 1993) and we found a novel candidate metastasis-associated gene *mta1* (Toh et al, 1994, 1995). Using the same or a similar method, several metastasis-associated genes have thus been isolated, including *nm23*, *mts-1*, WDNM1, WDNM2, elongation factor 1α, stromelysin 3 and pGM21 (Dear et al, 1988, 1989; Steeg et al, 1988; Ebralidze et al, 1989; Basset et al, 1990; Phillips et al, 1990; Taniguchi et al, 1992). With the exception of a few of these genes, such as *nm23* and stromelysin 3 (Steeg et al, 1988; Basset et al, 1990), however, few studies have examined the relationship between the expression of the genes in human carcinoma tissues and their clinicopathological features.

Our previous study showed that the mRNA expression level of *mta1* gene was fourfold higher in the highly metastatic mammary adenocarcinoma cell line MTLn3 than in the non-metastatic line MTC.4. The mRNA expression levels of the human homologue of this gene also correlated with metastatic potential in several human breast cancer metastatic systems. The *mta1* gene encodes a protein of the molecular mass of ~80 kDa and the correlation observed at mRNA level was also confirmed at the protein level. Although the function of *mta1* or its human counterpart *MTA1* gene product remains unknown, the deduced amino acid sequence suggests that the *mta1*/*MTA1* protein might be involved in a signal transduction pathway or regulation of gene expression (Toh et al, 1994, 1995). To determine whether the *mta1*/*MTA1* gene plays a role in cancers other than those of the breast, we recently examined the expression of the *MTA1* gene in surgically resected specimens of colorectal and gastric carcinomas and their corresponding normal mucosa tissues, and we found that overexpression of the *MTA1* gene was related to degree of invasion and lymphatic metastasis in these carcinomas (Toh et al, 1997). We have now extended our research to oesophageal carcinomas and have found a correlation between *MTA1* gene overexpression and tumour invasiveness.

*Received 16 April 1998*
*Revised 10 June 1998*
*Accepted 13 July 1998*

*Correspondence to:* Y Toh, Division of Pathology, Clinical Research Institute, National Kyushu Cancer Center, 3-1-1 Notame, Minami-ku, Fukuoka 811-1395, Japan

### MATERIALS AND METHODS

### Surgical specimens and isolation of total cellular RNA

Oesophageal carcinomas and paired normal mucosa were resected and snap-frozen in liquid nitrogen, immediately after surgical resection. The carcinoma tissue samples were obtained from a portion of the resected specimens after excluding necrotic or ulcerated parts and normal adjacent tissues. Specimens were stored at –80°C until the total RNA was extracted. Forty-seven oesophageal carcinomas along with their corresponding normal mucosa were obtained at Kyushu University Hospital and its facilitated hospitals in Japan during the 2-year period from 1995 to 1997.

Total cellular RNA was isolated from carcinomas and normal epithelia according to the method described by Chomzynski and Sacchi (1987) using Isogen (Nippon Gene, Tokyo, Japan) containing phenol and guanidine isothionate in a monophasic solution.

### cDNA synthesis by reverse transcription (RT) and quantitative RT-polymerase chain reaction (RT-PCR)

The methods for cDNA synthesis and optimization of quantitative RT-PCR for clinical samples were described in detail in our previous paper (Toh et al, 1997). The relative amounts of *MTA1* expression were normalized to glyceraldehyde-3-phosphate dehydrogenase (GAPDH) expression, and the *MTA1* expression in the carcinoma tissue (T) was compared with that in the corresponding normal mucosa (N). The tumour/normal ratio of *MTA1* expression (T/N ratio) in each case was calculated. To minimize variation, each PCR was performed in triplicate and the average expression of *MTA1* was compared between the carcinoma and the normal mucosa.

### Statistical analysis

All data regarding the clinical and histopathological variables were stored in a Macintosh computer. The Stat View program (Abacus Concepts, Berkeley, CA, USA) was used for all statistical analyses, using the chi-squared test and Fisher's exact probability test. A *P*-value less than 0.05 was judged to be statistically significant.

### RESULTS

To quantitate the relative levels of *MTA1* gene expression by RT-PCR and to compare the expression in paired carcinoma and normal epithelium samples, we previously determined the optimal conditions for proportional production of PCR product with amount of RNA template and the number of PCR cycles (Toh et al, 1997). Briefly, the result for *MTA1* indicates a linear relationship between the amount of PCR product and the number of PCR cycles between 30 and 34. Additional cycles reduced the linearity of the amplification response, whereas with fewer cycles the sensitivity of the PCR method decreased. This linear response was observed with three different starting amounts (0.5, 1.0 and 2.0 µg) of total RNA. Therefore, in this study, 32 cycles of amplification and 1.0 µg of total RNA were used for the RT-PCR analysis of *MTA1* gene expression. Similar experiments were performed for the RT-PCR of GAPDH mRNA, and the optimal conditions were determined to be 25 cycles of amplification and 1.0 µg of total RNA.

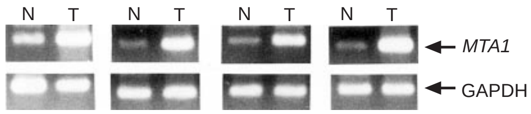

**Figure 1** *MTA1* mRNA expression in representative cases of oesophageal carcinomas as determined by the RT-PCR method. T represents tumour tissue and N represents the corresponding normal mucosa tissue. GAPDH is used as an internal control

**Table 1** Clinicopathological features of oesophageal carcinomas with regard to the relative expression of *MTA1* mRNA

| Variables | Relative expression of *MTA1* mRNA: Tumor/normal ≥2 (*n*=16) | Tumor/normal <2 (*n*=31) | *P*-values |
|---|---|---|---|
| Age (years) | 61.5±10.4 | 64.6±10.9 | 0.346 |
| Sex | | | |
| Male/female | 14:2 | 27:4 | 0.969 |
| Location | | | 0.760 |
| Upper third | 2 | 3 | |
| Middle third | 10 | 17 | |
| Lower third | 4 | 11 | |
| Histology | | | 0.777 |
| Well differentiated SCC[a] | 4 | 7 | |
| Moderately differentiated SCC | 8 | 18 | |
| Poorly differentiated SCC | 4 | 5 | |
| Small-cell carcinoma | 0 | 1 | |
| Depth of invasion | | | 0.031[c] |
| Within the wall | 4 | 18 | |
| Invaded into adventitia/ neighbouring structures | 12 | 13 | |
| Lymph node metastasis | | | 0.030[c] |
| Negative | 5 | 20 | |
| Positive | 11 | 11 | |
| Stage[b] | | | 0.121 |
| 0+I+II | 6 | 19 | |
| III+IV | 10 | 12 | |
| Lymphatic involvement | | | 0.081 |
| Negative | 5 | 18 | |
| Positive | 11 | 13 | |
| Vascular involvement | | | 0.337 |
| Negative | 8 | 20 | |
| Positive | 8 | 11 | |

[a]Differentiated squamous cell carcinoma; [b]the criteria used in this study are based on the rules established by the Japanese Society for Oesophageal Disease (1976); [c]statistically significant.

Having confirmed the reproducibility of our quantitative RT-PCR assay for *MTA1* gene expression, we analysed the relative expression of *MTA1* mRNA in the 47 surgically resected oesophageal carcinomas and compared them with the corresponding normal mucosa tissues. All of the normal oesophageal mucosa expressed *MTA1* mRNA in relatively low amounts. Using densitometric analysis of the PCR products, we estimated overexpression (T/N ratio ≥ 2) of the *MTA1* mRNA compared with the normal mucosa in 16 out of 47 cases (34.0%), whereas, in the remaining 31 cases, the expression of *MTA1* mRNA was similar in the carcinoma and normal mucosa tissues. The representative cases that overexpressed *MTA1* mRNA are shown in Figure 1.

*© Cancer Research Campaign 1999*

The relationship between expression of the *MTA1* gene and clinicopathological data of the oesophageal carcinomas was examined. In the oesophageal carcinoma cases, we arbitrarily divided the samples into two groups depending on the level of expression of the *MTA1* gene in the tumour samples (T/N ratio ≥ 2 and T/N ratio < 2) (Table 1). Clinicopathological studies demonstrated that 12 out of 16 oesophageal carcinomas overexpressing this gene (T/N ratio ≥ 2) invaded into the adventitia or the neighbouring structures, whereas 13 out of 31 remaining tumours (T/N ratio < 2) were restricted in the proper muscle layer ($P < 0.05$). Furthermore, *MTA1*-overexpressing tumours had a significantly higher rate of lymph node metastasis ($P < 0.05$) and tended to have a higher rate of lymphatic involvement ($P = 0.081$). No statistically significant differences were observed in age, sex, location of the main tumour, histological differentiation, stage and vascular involvement.

### DISCUSSION

About 85% of patients with oesophageal carcinomas are in an advanced stage of the disease at the time of the operation (Sugimachi et al, 1994), and approximately 40% of these patients have either local tumour invasion into the neighbouring structures or lymph node metastases that preclude complete removal of the cancers (Sugimachi et al, 1994). Furthermore, the histological findings of lymph node metastasis and the presence of vascular or lymphatic invasion by cancer cells have been widely accepted as unfavourable prognostic factors (Sugimachi et al, 1988). Therefore, to improve the outcome of the treatment of this disease, it may be necessary not only to diagnose this disease at an early stage but also to understand the molecular mechanisms of invasion and metastasis of oesophageal carcinomas.

Here, we showed that *MTA1* gene could be another candidate factor that is associated with invasion and metastasis of oesophageal carcinomas. Previously, we found that *MTA1* mRNA was also overexpressed in other tumour tissues in comparison to the normal mucosal tissues (T/N ratio ≥ 2), e.g. in 38.9% of colorectal carcinomas and 38.2% of gastric carcinomas. The colorectal and gastric carcinoma tumours that overexpressed *MTA1* mRNA also showed deeper invasion and higher rates of lymph node metastasis (Toh et al, 1997). That overexpression of the *MTA1* mRNA was significantly correlated with tumour invasiveness in three independent carcinoma types strongly suggests the possibility that *MTA1* is one of the key molecules associated with tumour invasion and metastasis.

Although the exact function of the *mta1*/*MTA1* gene product is still unknown, its deduced amino acid sequence suggests that this protein is involved in a signal transduction pathway (Toh et al, 1994; 1995). The protein motifs of the *MTA1* gene product suggested that there are potential phosphorylation sites for tyrosine kinases, protein kinase C and casein kinase II. In addition, a proline-rich amino acid sequence (LPLRPPPPAP) exists at the carboxy-terminal extremity that completely matches the consensus sequence for a *src* homology 3 domain (SH3)-binding site (Ren et al, 1993; Yu et al, 1994). SH3 domains are considered important in protein–protein interactions in signal transduction pathways (Pawson and Gish, 1992), and they are often associated with cytoskeletal components (Bar-Sagi et al, 1993) as well as being involved in other protein–protein interactions (Seidel-Dugan et al, 1992).

Recently, Paterno et al (1997) reported a novel, developmentally regulated, immediate early gene named *er* [1] which is activated by fibroblast growth factor in *Xenopus* embryos. The predicted ER1 amino acid sequence (accession number AF015454) contains three regions of similarity to *mta1*/*MTA1* gene product which are in turn similar to the *MTA1*-like sequence found in a *C.e legans* protein (accession number U41264). The *er*[1] gene has been shown to be a potent transcription factor and it also contains a SH3-binding domain. Although the *er*[1] gene does not seem to be a *Xenopus* homologue of the rat *mta*[1] gene, the data suggest that they are related members of a gene family with the same or similar functions. Indeed, the amino acid sequence of *mta1*/*MTA1* contains a Zinc-finger motif which is important in some transcription factors. Therefore, it may be possible that *mta1*/*MTA1* protein regulates the expression of a set of proteins which are associated with invasion and metastasis of oesophageal carcinoma cells. For example, Shima et al (1992) reported that matrix metalloproteinase 2 (72 kDa type IV collagenase) and stromelysin were expressed in a portion of the oesophageal carcinomas and they found a good correlation between expression of the matrix metalloproteinase and lymph node metastasis and vascular invasion. Using immunohistochemistry, Kadowaki et al (1994) examined the expressions of E-cadherin and α-catenin in oesophageal squamous cell carcinomas. They found that the reduction of α-catenin and E-cadherin expression was significantly associated with increased invasiveness and lymph node metastasis. Thus, it is of interest to investigate the relationship between the expression of metastasis-associated proteins described above and that of *mta1*/*MTA1* gene product in oesophageal carcinomas. For this purpose, immunohistochemical co-localization of these proteins in resected oesophageal specimens and artificial overexpression *mta1*/*MTA1* gene in oesophageal carcinoma cell lines followed by in vitro assays for invasion, motility and adhesion will be required.

In conclusion, our studies have shown that the expression of the *MTA1* gene is closely related to the invasiveness of oesophageal carcinomas as well as gastric and colorectal carcinomas. The novel *MTA1* gene could thus potentially provide new information on the mechanism of invasion and metastasis of the gastrointestinal cancers. To elucidate the association of this gene with invasion and metastasis, transfection studies using sense and antisense *mta1*/*MTA1* cDNA expression plasmids will be necessary. In addition, it is also important to examine *MTA1* mRNA expression in various other human cancers, and future studies should therefore include breast, lung and other cancer specimens. Such studies are presently in progress in our laboratories.

### ACKNOWLEDGEMENTS

This work was supported in part by Research Grant of the Princess Takamatsu Cancer Research Fund and a Grant-in-Aid for Scientific Research from the Ministry of Education, Science and Culture of Japan to YT and a grant from the US National Institute of Health to GLN.

### REFERENCES

Bar-Sagi D, Rotin D, Batzer A, Mandiyan V and Schlessinger J (1993) SH3 domains direct cellular localization of signaling molecules. *Cell* **74**: 83–91

Basset P, Bellocq JP, Wolf C, Stoll I, Hutin P, Limacher JM, Podhajcer OL, Chenard MP, Rio MC and Chambon P (1990) A novel metalloproteinase gene specifically expressed in stromal cells of breast carcinomas. *Nature* **348**: 699–704

© Cancer Research Campaign 1999

Chomczynski P and Sacchi N (1987) Single-step method of RNA isolation by acid guanidinium thiocyanate–phenol–chloroform extraction. *Anal Biochem* **162**: 156–159

Dear TN, Ramshaw IA and Kefford RF (1988) Differential expression of a novel gene, WDNM1, in nonmetastatic rat mammary adenocarcinoma cells. *Cancer Res* **48**: 5203–5209

Dear TN, McDonald DA and Kefford RF (1989) Transcriptional down-regulation of a rat gene, WDNM2, in metastatic DMBA-8 cells. *Cancer Res* **49**: 5323–5328

Ebralidze A, Tulchinsky E, Grigorian M, Afanasyeva A, Senin V, Revazova E and Lukanidin E (1989) Isolation and characterization of a gene specifically expressed in different metastatic cells and whose deduced gene product has a high degree of homology to a $Ca^{2+}$-binding protein family. *Genes Develop* **3**: 1086–1093

Hollstein MC, Metcalf RA, Welsh JA, Montesano R and Harris CC (1990) Frequent mutation of the p53 gene in human oesophageal cancer. *Proc Natl Acad Sci USA* **87**: 9958–9961

Japanese Society for Oesophageal Disease (1976) Guidelines for the clinical and pathologic studies on carcinoma of the oesophagus. *Jpn J Surg* **6**: 69–78

Kadowaki T, Shiozaki H, Inoue M, Tamura S, Oka H, Doki Y, Iihara K, Matsui S, Iwazawa T, Nagafuchi A, Tsukita S and Mori T (1994) E-cadherin and alpha-catenin expression in human oesophageal cancer. *Cancer Res* **54**: 291–296

Liotta LA, Steeg PS and Stetler-Stevenson WG (1991) Cancer metastasis and angiogenesis: an imbalance of positive and negative regulation. *Cell* **64**: 327–336

Lu SH, Hsieh LL, Luo FC and Weinstein IB (1988) Amplification of the EGF receptor and c-myc genes in human oesophageal cancers. *Int J Cancer* **42**: 502–505

Nakagawa H, Zukerberg L, Togawa K, Meltzer SJ, Nishihara T and Rustgi AK (1995) Human cyclin D1 oncogene and oesophageal squamous cell carcinoma. *Cancer* **76**: 541–549

Nicolson GL (1988) Cancer metastasis: tumor cell and host organ properties important in metastasis to specific secondary sites. *Biochim Biophys Acta* **948**: 175–224

Paterno GD, Yu L, Luchman HA, Ryan PJ and Gillespie LL (1997) cDNA cloning of a novel, developmentally regulated immediate early gene activated by fibroblast growth factor and encoding a nuclear protein. *J Biol Chem* **272**: 22591–25595

Pawson T and Gish GD (1992). SH2 and SH3 domains: from structure to function. *Cell* **71**: 359–362

Pencil SD, Toh Y and Nicolson GL (1993) Candidate metastasis-associated genes of the rat 13762NF mammary adenocarcinoma. *Breast Cancer Res Treat* **25**: 165–174

Phillips SM, Bendall AJ and Ramshaw IA (1990) Isolation of gene associated with high metastatic potential in rat mammary adenocarcinomas. *J Natl Cancer Inst* **82**: 199–203

Ren R, Mayer BJ, Cicchetti P and Baltimore D (1993) Identification of a ten-amino-acid proline-rich SH3 binding site. *Science* **259**: 1157–1161

Seidel-Dugan C, Meyer BE, Thomas SM and Brugge JS (1992) Effects of SH2 and SH3 deletions on the functional activities of wild-type and transforming variants of c-Src. *Mol Cell Biol* **12**: 1835–1845

Shima I, Sasaguri Y, Kusukawa J, Yamana H, Fujuta H, Kakegawa T and Morimatsu M (1992) Production of matrix metalloproteinase-2 and metalloproteinase-3 related to malignant behavior of esophageal carcinoma. A clinicopathologic study. *Cancer* **70**: 2747–2753

Steeg PS, Bevilacqua G, Kopper L, Thorgeirsson UP, Talmadge JE, Liotta LA and Sobel ME (1988) Evidence for a novel gene associated with low tumor metastatic potential. *J Natal Cancer Inst* **80**: 200–204

Sugimachi K, Matsuoka H, Ohno S, Mori M and Kuwano H (1988) Multivariate approach for assessing the prognosis of clinical oesophageal carcinoma. *Br J Surg* **75**: 1115–1118

Sugimachi K, Watanabe M, Sadanaga N, Ikebe M, Kitamura K, Mori M and Kuwano H (1994) Recent advances in the diagnosis and surgical treatment of patients with carcinoma of the oesophagus. *J Am Coll Surg* **178**: 363–368

Taniguchi S, Miyamoto S, Sadano H and Kobayashi H (1992) Rat elongation factor 1 alpha: sequence of cDNA from a highly metastatic *fos*-transferred cell line. *Nucleic Acids Res* **19**: 6949

Toh Y, Pencil SD and Nicolson GL (1994) A novel candidate metastasis-associated gene, *mtal*, differentially expressed in highly metastatic mammary adenocarcinoma cell lines. cDNA cloning, expression, and protein analyses. *J Biol Chem* **269**: 22958–22963

Toh Y, Pencil SD and Nicolson GL (1995) Analysis of the complete sequence of the novel metastasis-associated candidate gene, *mta1*, differentially expressed in mammary adenocarcinoma and breast cancer cell lines. *Gene* **159**: 97–104

Toh Y, Oki E, Oda S, Tokunaga E, Ohno S, Maehara Y, Nicolson GL and Sugimachi K (1997) Overexpression of the *MTA1* gene in gastrointestinal carcinomas: correlation with invasion and metastasis. *Int J Cancer* **74**: 459–463

Yu H, Chen JK, Feng S, Dalgarno DC, Brauer AW and Schreiber SL (1994) Structural basis for the binding of proline-rich peptides to SH3 domains. *Cell* **76**: 933–945

*© Cancer Research Campaign 1999*

